# Structure-Activity Relationships of the Imidazolium Compounds as Antibacterials of *Staphylococcus aureus* and *Pseudomonas aeruginosa*

**DOI:** 10.3390/ijms22157997

**Published:** 2021-07-27

**Authors:** Łukasz Pałkowski, Maciej Karolak, Jerzy Błaszczyński, Jerzy Krysiński, Roman Słowiński

**Affiliations:** 1 Department of Pharmaceutical Technology, Faculty of Pharmacy, Nicolaus Copernicus University, Jurasza 2, 85-094 Bydgoszcz, Poland; maciej.karolak@cm.umk.pl (M.K.); jerzy.krysinki@cm.umk.pl (J.K.); 2 Institute of Computing Science, Poznań University of Technology, Piotrowo 2, 60-965 Poznań, Poland; Jerzy.Blaszczynski@cs.put.poznan.pl (J.B.); roman.slowinski@cs.put.poznan.pl (R.S.); 3 Systems Research Institute, Polish Academy of Sciences, Newelska 6, 01-447 Warsaw, Poland

**Keywords:** gemini-imidazolium chlorides, antimicrobial activity, molecular descriptors, SAR, dominance-based rough set approach (DRSA)

## Abstract

This paper presents the results of structure–activity relationship (SAR) studies of 140 3,3’-(α,ω-dioxaalkan)bis(1-alkylimidazolium) chlorides. In the SAR analysis, the dominance-based rough set approach (DRSA) was used. For analyzed compounds, minimum inhibitory concentration (MIC) against strains of *Staphylococcus aureus* and *Pseudomonas aeruginosa* was determined. In order to perform the SAR analysis, a tabular information system was formed, in which tested compounds were described by means of condition attributes, characterizing the structure (substructure parameters and molecular descriptors) and their surface properties, and a decision attribute, classifying compounds with respect to values of MIC. DRSA allows to induce decision rules from data describing the compounds in terms of condition and decision attributes, and to rank condition attributes with respect to relevance using a Bayesian confirmation measure. Decision rules present the most important relationships between structure and surface properties of the compounds on one hand, and their antibacterial activity on the other hand. They also indicate directions of synthesizing more efficient antibacterial compounds. Moreover, the analysis showed differences in the application of various parameters for Gram-positive and Gram-negative strains, respectively.

## 1. Introduction

Cationic surfactants, including bis-imidazolium chlorides (also called gemini-compounds), are able to lower the surface tension while keeping good antimicrobial activity. This activity is a result of the interaction of the cationic surfactant with the cell membrane of the microorganism, which leads to the disintegration of membrane structure, the degradation of proteins and nucleic acids, and cell death. The type of interactions and the mechanism of antimicrobial effect depend largely on the surfactant concentration, pH, temperature, type of microorganism, or concentration of other ions. Quaternary ammonium salts (containing quaternary nitrogen in its molecule) are particularly active against Gram-positive bacteria. In contrast, Gram-negative bacteria are less susceptible to those salts, which results in a tendency to acquire resistance to this group of compounds. The high affinity of the quaternary salts to the biological membranes also results in an effective antifungal activity, and ability to inhibit the growth of yeast by inhibiting H+-ATPase [1,2,3].

*Staphylococcus aureus* (SAU) and *Pseudomonas aeruginosa* (PAE) are microbial strains with different susceptibility to antibiotics, disinfectants, and antiseptics. This is due to the different construction of the cell wall. Hence, the parameters affecting antibacterial activity of chemical compounds on these microbes are different.

Humans are a natural reservoir of SAU (Gram-positive cocci). Humans colonized with *S. aureus* are at increased risk for subsequent infections. Rates of staphylococcal colonization are high among patients with type 1 diabetes, intravenous drug users, patients undergoing hemodialysis, surgical patients, and patients with the acquired immunodeficiency syndrome. Patients with qualitative or quantitative defects in leukocyte function are also at increased risk for staphylococcal disease [4].

PAE is a common environmental Gram-negative bacillus which acts as an opportunistic pathogen under several circumstances. The ubiquitous occurrence of PAE in the environment is due to several factors, including its abilities to colonize multiple environmental niches and to utilize many environmental compounds as energy sources. Nearly all clinical cases of PAE infection can be associated with the compromise of host defense. While many cases of PAE infection can be attributed to general immunosuppression, such scenarios predispose the host to a variety of bacterial and fungal infections, and therefore do not yield information which is specific to the pathogenesis of PAE. In this respect, three of the more informative human diseases caused by PAE are: bacteremia in severe burn victims, chronic lung infection in cystic fibrosis patients, and acute ulcerative keratitis in users of extended-wear soft contact lenses [5,6].

Given the limited number of suitable and effective antimicrobial agents, together with increasing drug resistance of the pathogens, it is important that new classes of anti-infectives are discovered. 

The aim of the work was to understand the structure–activity relationship (SAR) of 140 3,3′-(α, ω-dioxaalkan)bis(1-alkylimidazolium) chlorides on SAU and PAE strains with different susceptibility to antimicrobial compounds. Moreover, we wanted to discover and compare parameters that are of the highest impact on bacteriostatic action against SAU and PAE, being completely different microorganisms. A large group of homologous series of compounds enables the identification of the factors affecting the antimicrobial activity of the bis-imidazolium chlorides. 

In the structure–activity relationship study (SAR), a modified method, based on a rough set theory, was employed. The dominance-based rough set approach (DRSA) has been proposed by Greco, Matarazzo, and Słowiński [7,8]. DRSA extends rough set theory proposed by Pawlak [9] and follows the suggestion formulated by Słowiński in [10] towards reasoning about decision situations with background knowledge about ordinal evaluations of objects from a universe, and about monotonic relationships between these evaluations. Precisely, the monotonic relationships are assumed between evaluation of objects on condition attributes and their assignment to decision classes. The monotonic relationships are also interpreted as monotonicity constraints, because the better the evaluation of an object, the better the decision class the object is assigned to should be. For this reason, classification problems of this kind are called ordinal classification problems with monotonicity constraints. Many real-world classification problems fall into this category [11]. Typical examples are multiple criteria sorting and decision under uncertainty, where the order of value sets of attributes corresponds to increasing or decreasing order of preference of a decision maker.

It is worth stressing, however, that DRSA can also be used in data analysis of non-ordinal problems, that is, problems with no background knowledge about ordinal evaluations of objects, after an easy pre-processing of the input data [12]. It then gives more concise decision rules than the usual induction techniques designed for non-ordinal classification, without recurring to a pre-discretization of numerical attributes.

In DRSA, lower approximation of a union of ordered decision classes contains only consistent objects. Such a lower approximation is defined as a sum of dominance cones that are subsets of the approximated union. In practical applications, however, such a strong requirement may result in relatively small (and even empty) lower approximations. Therefore, several variants of DRSA have been proposed, relaxing the condition for inclusion of an object to the lower approximation. Variable consistency dominance-based rough set approaches (VC-DRSA) include lower approximations of objects that are sufficiently consistent, according to different measures of consistency.

## 2. Results and Discussion

Quaternary ammonium chlorides with a hydrophobic alkyl chain from decyl to hexadecyl have been used for years as antimicrobial agents. It has been demonstrated that the MIC values of the bis-quaternary ammonium compounds are lower for bacterial strains that are Gram-positive than that of Gram-negative bacteria [13]. This is due to the fact that the cell walls of Gram-negative microorganisms is less permeable than that of Gram-positive microorganisms [14]. Similar results were observed for the tested gemini-imidazolium chlorides. In certain homologous series of strains, the MIC values for SAU (Gram-positive bacteria) are lower than the PAE (Gram-negative bacteria). This relationship becomes more apparent with increasing length of n-linker and R-substituent. Similar conclusions can be found in [15].

**Figure 1:**
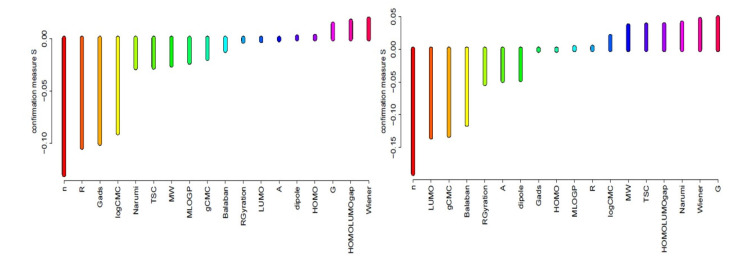
Predictive attribute confirmation calculated for MIC decision classes SAU “weak” (left) and “good” (right).

**Figure 2:**
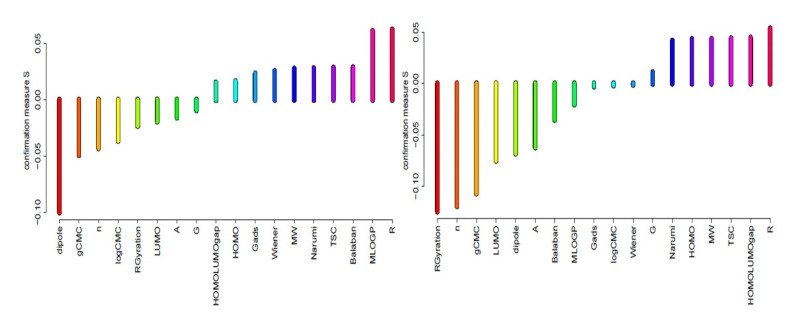
Predictive attribute confirmation calculated for MIC decision classes PAE “weak” (left) and “good” (right).

Rankings of condition attributes for different sets of objects have been constructed on the basis of values of s-confirmation measures, which indicates the impact of condition attributes for object classification. Attributes having the highest confirmation measures are important from the point of view of the proper assignment of objects (chemicals) for the appropriate class of antimicrobial activity. Results of estimation of predictive confirmation of all attributes (structure, surface active, and molecular) in rules induced for classes good and weak are presented in Figure 1 and Figure 2. 

The analysis of Figure 1 and Figure 2 indicates that in the group of 3,3’-(α, ω-dioksaalkan)bis(1-alkylimidazolium) chlorides, the most important in relation to the SAU in class 1 (good) are the following conditional attributes: value of surface excess (G), Wiener index, Narumi topological index, HOMO–LUMO gap, TSC, and MW. Assigning to the third class (weak) is determined by highest occupied molecular orbital, electric dipole moment, molecular area of single particle, and lowest unoccupied molecular orbital. However, values of confirmation measures are very low. On the other hand, with respect to the PAE strain in class 1 (good), the most important are: the length of R, the energy difference between the HOMO and LUMO, TSC, MW, highest occupied molecular orbital, and Narumi index. Nevertheless, assigning to class 3 is decided in this case by the length of R, Moriguchi octanol-water partition coefficient, Balaban index, TSC, Narumi index, and molecular weight.

The obtained decision rules are structured in accordance with the decreasing number of objects that support them. In addition, rules have been characterized by the strength, which means the ratio of the number of objects that support the rule to the total number of objects in the collection, as well as the confirmation measure, *s*, which informs about the suitability of the rules in distinguishing the correct classification of objects. Strong decision rules supported by a large number of objects and a high measure of confirmation, *s*, provide information about the most important features leading to chemical compounds with strong or weak antimicrobial properties. Decision rules thereby do not contain any irrelevant features that obscure the picture of cause and effect between a condition and decision attributes.

Table 1 and Table 2 include strong and relevant decision rules obtained for good and weak classes of chlorides. These are rules selected from the set of all minimal decision rules induced from the information table processed by DRSA.

We do not induce rules for the class “medium” since these rules are not interesting from the view point of SAR analysis (it is more important to know the features of chlorides with definitely good or weak antimicrobial properties). Note, however, that the presence of chlorides from the “medium” class is important in the rule induction process. The rules with conclusion “good” discriminate chlorides with “good” antimicrobial properties from those chlorides which have “medium” or “weak” properties (analogously for rules with conclusion “weak”).

Table 1 presents the 10 most important decision rules for the SAU strain. In the class of good antimicrobial activity, only 3 condition attributes are present: the length of R, value of surface excess, and Wiener index. Compounds active against the SAU strain would therefore have 7–11 atoms of carbon in the R-substituent. The value of surface excess should be in the range of 2.47–2.71 mol/m^2^. Moreover, the Wiener index should be greater than 9.67. Weak anti-SAU compounds are described by the value of surface tension at CMC greater than or equal to 50.3 mN/m, value of surface excess greater than or equal to 2.57 mol/m^2^, and Wiener index lower than 8.56. The value of the HOMO–LUMO gap is also of great importance, with the value lower than −0.16.

Rules obtained for the PAE strain (Table 2) present that for high activity against this microorganism, the length of R-substituent (R ≥ 6) and n-spacer (n ≥ 6; however, supported by 1 decision rule only) are important. The value of molecular area of a single particle (A ≤ 83 × 10^−20^ m^2^) should also be taken into consideration. Single rules also inform about the values of molecular weight, HOMO–LUMO gap, Balaban index, and total structure connectivity index. Gemini-chlorides with n-spacer ≤ 7 and R-substituent equal to 2 or 3 would be of low activity against PAE. Moreover, condition attributes: free energy of adsorption of molecule, Moriguchi octanol-water partition coefficient, HOMO–LUMO gap, Balaban index, and total structure connectivity index would also decide about low antipseudomonal action.

Previous research in this area [8,16,17] has shown that good antimicrobial activity for a group of analyzed gemini chlorides is related to n-spacer equal to or longer than 6 atoms of carbon. Moreover, we discovered more features in a strong relationship with a good antifungal activity, regarding *Candida albicans* strains. Those are not only the length of substituents in a moiety, but also logCMC and **γ**CMC, Moriguchi octanol-water partition coefficient, the energy difference between the HOMO and LUMO, Balaban index, Narumi topological index, and total structure connectivity index. Those parameters should be taken into consideration when planning synthesis of a new gemini chloride with a high anti-*Candida albicans* activity.

When we consider the SAU strain, we previously found that surface-active properties strongly influence the antimicrobial activity. Structural attributes are less significant. Moreover, the value of R-chain for active compounds ranges from 6 to 11 (the alkyl chain longer than hexyl and shorter than the undecyl one), −logCMC values are in the range (2.71–3.13), values of surface tension at critical micelle concentration are below 46.4, values of surface excess are below 2.42, and values of free energy of adsorption of molecule are >24.6. 

In [18], the authors point out that compounds having low values of surface tension (37–41 mN/m) exhibit the highest antimicrobial activity with respect to SAU. It is also connected to the length of R, and thus the increase of the hydrophobicity of the particles. This is particularly important in the case of Gram-positive bacteria as the lipophilicity of the molecule facilitates the penetration of the surfactant into the interior of a bacterial cell.

It was also observed that the decrease in the hydrophilicity of the particles leads to a decrease of the surface excess as well as the reduction of the Gads. However, the area occupied by the molecule of the surfactant adsorbed at the interface increases with the number of carbon atoms in the alkyl chain of the surfactant molecule. This results in an increase in the activity of the compounds against Gram-positive bacteria [19,20,21].

The incorporation of molecular descriptors in SAR analysis for SAU and PAE strains was not considered so far. Wiener index, being a part of all strong decision rules for SAU, is a two-dimensional molecular descriptor. It is derived from topological representation of compound structure by applying mathematical operators directly to graph-theoretical matrices. WI is defined as the sum of the topological distances between all the atom pairs, but it is commonly calculated as the half-sum of the distance matrix entries [22,23]. The interpretation of WI in the context of induced decision rules may be rather difficult. According to a few papers, it can be physically related to molecular volume, since WI value increases for large molecules. By its algorithm, each bond makes a contribution equal to the product of the number of atoms on each side of the bond considered [24]. In SAR analysis for SAU, it can be interpreted as the higher the distances between atoms in molecules, the better antibacterial activity this compound presents.

### Comparison of DRSA with Alternative Methods

In order to assess the relative predictive ability of DRSA, we performed a repeated cross-validation test involving VC-bagging (DRSA), Random Forest, and Logistic Regression methods. The last two methods are considered as off-the-shelf methods able to obtain very good predictive accuracy results.

The results of this comparative test are presented in Table 3. One can observe that the accuracy of classification was high for both classes of activity. Nevertheless, better results were obtained for weak antimicrobial compounds (class 3). To validate the results obtained with VC-bagging, we performed the same type of stratified cross-validation with Random Forest and Logistic Regression implemented in the WEKA toolkit [25]. These results show that VC-bagging produces comparable results to Random Forest. Both VC-bagging and Random Forest produce better results than Logistic Regression.

## 3. Materials and Methods

### 3.1. Chemical Compounds

**Figure 3:**
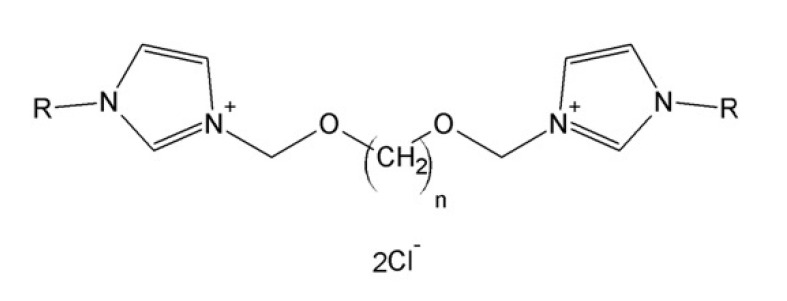
Chemical structure of analyzed compounds.

For the sake of the SAR analysis, 140 bis-imidazolium quaternary chlorides were synthesized and examined. Figure 3 presents the basic chemical structure of studied compounds. The general procedure for the synthesis of compounds was described previously in [26]. 

### 3.2. Structural Parameters

The chemical structure of chlorides, presented in Figure 3, was described by the number of carbon atoms in n-spacer (n), and the number of carbon atoms in R-substituent (R). The length of n-linkers and R-substituents in the structures of the tested compounds and their encoding in the information systems are presented in Table 4.

### 3.3. Surface-Active Parameters

Properties of analyzed gemini-imidazolium compounds were described by: critical micelle concentration (CMC; mol/L; calculated as a logCMC value), value of surface tension at CMC (γCMC; mN/m), value of surface excess (Γ (G) × 10^6^; mol/m^2^), molecular area of a single particle (A; A × 10^−20^ m^2^), and free energy of adsorption of molecule (∆G_ads_; kJ/mol). Details of the surface-active properties’ evaluation can be found in [14].

### 3.4. Molecular Descriptors

The geometry of compounds was optimized using quantum mechanical Density Functional Theory (DFT), using the B3LYP method with Pople’s 6–31G basis set. Optimization was performed in water solvent with the use of the Polarizable Continuum Model. Calculations were performed employing Gaussian 09, Revision D.02 (Gaussian, Inc., Wallingford, CT, USA), on a supercomputer cluster nodes with 12-core Intel^®^ Xeon^®^ E5 v3 2.3 GHz processors.

Molecular descriptors considered in the analysis were determined using quantum mechanical calculations and Dragon 7.0 software (Talete, Milan, Italy). Descriptors with missing or constant values were excluded from the analysis. Among the numerous descriptors derived, the following ones were chosen a priori for SAR analysis: Moriguchi octanol-water partition coefficient (MLOGP), Balaban index (BI), Narumi topological index (NTI), total structure connectivity index (TSC), Wiener index (WI; numerical parameters characterizing compounds’ topology), highest occupied molecular orbital (HOMO), lowest unoccupied molecular orbital (LUMO), HOMO–LUMO gap (HL gap; the energy difference between the HOMO and LUMO), dipole (dip; electric dipole moment), radius of gyration (ROG; the root mean square distance of the entities’ parts from either its center of gravity or a given axis), and molecular weight (MW) of compounds.

Descriptors chosen were the ones with the highest informational load in the selection process based on the expert experience. Moreover, it was assumed that descriptors should be interpretable, and possible to be used as guidance for future synthesis of new, active compounds. Nevertheless, it is important to bear in mind the limitations of this method of selecting descriptors, in particular when drawing conclusions.

### 3.5. Antimicrobial Activity

The antimicrobial activity of synthesized chlorides was tested against: *Staphylococcus aureus* ATCC 25213 and *Pseudomonas aeruginosa* ATCC 27853. Details of MIC determination can be found in [14].

### 3.6. Dominance-Based Rough Set Approach to SAR Study

Data that concern antibacterial activity of n-alkyl-bis-*N*-alkoxy-*N*-alkyl imidazolium chlorides can be seen as classification data. Surface-active properties and molecular parameters of chlorides were used as condition attributes (independent variables). The decision attribute (dependent variable) is distinguishing the following classes of MIC: good, medium, and weak. To explain the class assignment in terms of condition attributes, we used the rough set concept, and its particular extension, called the dominance-based rough set approach (DRSA) [27,28].

DRSA was chosen for SAR analysis because it is able to deal with inconsistencies in the information system prior to induction of rules. Moreover, it handles global or local monotonic relationships between values of condition attributes and quality of the decision class. These characteristics of DRSA perfectly fit the type of data to be analyzed. Using this method, we obtain decision rules with ranges of values of condition attributes in particular classes of activity. Moreover, this method is able to discover synthetic rules that exhibit monotonic relationships between structure, surface-active properties, and molecular parameters of the compounds on the one hand, and their antimicrobial properties on the other hand. This method can handle heterogeneous information (qualitative and quantitative, ordered and non-ordered) and scales of preference (ordinal, cardinal), while classical methods consider only quantitative ordered evaluations, with rare exceptions.

#### 3.6.1. Information System

The information system (see Table 5) is the input data for SAR analysis of the chemical compounds. It includes (in rows) a set of objects (compounds) described by a set of attributes (in columns). The set of attributes is composed of condition attributes and two decision attributes, considered as two different classifications. In our case, condition attributes describe surface-active properties, structure of analyzed chlorides, and molecular parameters. The decision attributes concern antimicrobial properties of bis-quaternary imidazolium chlorides, distinguishing 3 classes of MIC values for SAU ATCC 25213 and PAE ATCC 27853. The classes of these two decision attributes are defined in the following way: class of good antimicrobial properties: MIC ≤ 0.02 mM/L, class of medium antimicrobial properties: 0.02 < MIC < 1 mM/L, and class of weak antimicrobial properties: MIC ≥ 1 mM/L. Values of MIC for activity classes were determined on the basis of antimicrobial activity of benzalkonium chloride and didecyldimethylammonium chloride, used as reference antimicrobials. Table 5 presents a part of the information system. A complete table presenting all 140 compounds may be found in the Supplementary Materials.

#### 3.6.2. Validation

The analyzed information system was divided randomly into a training and a test set, according to stratified cross-validation scheme, in a ratio of 4 to 1, i.e., 5-fold cross-validation [29]. Decision rules induced from the training set were checked on the validation set. The calculations were repeated 10,000 times.

#### 3.6.3. Decision Rules

The algorithm for induction of decision rules—VC-DomLEM—has been implemented as a software package called jMAF, based on the java Rough Set (jRS) library [30]. VC-DomLEM is applied on bootstrap samples obtained according to the VC-bagging scheme described in [31]. Decision rules represent the most important cause–effect relationships between values of condition attributes and the class assignment discovered from the information system.

The decision rules explain regularities found in the past experience and provide guidelines for synthesis of new compounds with good antimicrobial properties. The rules are characterized by various parameters, such as support (i.e., number of objects covered by a given rule), strength (i.e., the proportion of objects covered by the premise that they are also covered by conclusion), and confirmation (i.e., Bayesian confirmation measure that is quantifying the degree to which a premise provides evidence for a conclusion). Table 1 and Table 2 present only these attributes included in decision rules. 

#### 3.6.4. Attribute Relevance

We consider attribute relevance measures that satisfy the property of Bayesian confirmation. These measures take into account interactions between attributes present in the decision rules. In this case, the property of confirmation is related to quantification of the degree to which the presence of an attribute in the premise of a rule provides evidence for or against the conclusion of the rule [32,33].

## 4. Conclusions

The comparative analysis indicated that, from the predictive accuracy perspective, DRSA is positively compared to some well-known methods applied to SAR analysis. It also has other advantages, such as intelligible knowledge representation by monotonic decision rules, and useful ranking of the relevance of attributes.

The resulting decision rules can be regarded as valuable indications for the synthesis of new chemical compounds with expected high antimicrobial activity. This fact is acknowledged by the high correctness of the classification, as well as the high accuracy and precision of the presented method. Those rules may serve to accelerate the discovery of new active anti-infective agents. Ranges of values of structure and surface-active attributes and molecular descriptors in the condition parts of decision rules are important from the point of view of the synthesis of new chemical entities with good antimicrobial properties. 

This SAR study revealed that activity of gemini-imidazolium compounds against different microbial strains is dependent on different features (condition attributes). When SAU is taken into consideration, surface-active properties and molecular descriptors seem to predominate structural ones. However, when PAE is taken into account, chemical structure attributes are of greater importance than others.

## Figures and Tables

**Table 1:** Decision rules for SAU.

No.	Condition Attributes							Support	Strength	Confirmation
	n	R	γCMC	Γ 10^6^	∆G_ads_	HLgap	WI			
Decision Class “Weak”										
**1**			≥50.3	≥2.57			≤8.56	18	12.85	0.9230
**2**				≥2.57	≤23	≤−0.15	≤8.56	17	12.14	0.9583
**3**			≥52.1	≥2.57		≤−0.16	≤8.56	17	12.14	0.9230
**4**			≥53.4	≥2.57			≤7.46	17	12.14	0.9200
**5**						≤−0.16	≤8.43	17	12.14	0.8888
**Decision Class “Good”**										
**6**		≥7		≤2.47			≥10.49	41	29.28	0.7894
**7**		[6,7,8,9]		≤2.47			≥10.49	33	23.57	0.7500
**8**		≥7		≤2.47			≥9.67	25	17.85	0.6956
**9**		≤11		≥2.71			≥10.49	24	17.14	0.7500
**10**		≥7		≤2.46			≥10.49	24	17.14	0.6956

**Table 2:** Decision rules for PAE.

No.	Condition Attributes										Support	Strength	Confirmation
	n	R	γCMC	A × 10^20^	∆G_ads_	MlogP	MW	HLgap	BI	TSC			
Decision Class “Weak”													
**1**		≥2								≥0.21	32	0.2285	0.8947
**2**	≤7					≤3.025					32	0.2285	0.9000
**3**					≥22	≤3.025					27	0.1928	0.8181
**4**			≥51.1						≥1.33		19	0.1357	0.8571
**5**		≤3						≤−0.1746			19	0.1357	0.8571
**Decision Class “Good”**													
**6**		≥6		≤83							45	0.3214	0.8333
**7**		≥6						≥−0.1797		≥0.18	41	0.2928	0.7500
**8**	≥6	≥6		≤83							31	0.2214	0.7500
**9**		≥6		≤83					≤1.277		30	0.2142	0.7500
**10**		≥7					≤603				27	0.1928	0.7894

**Table 3:** Comparison of DRSA with alternative methods.

	VC-Bagging (DRSA)				Random Forest				Logistic Regression			
Validation Parameter	SAU		PAE		SAU		PAE		SAU		PAE	
	Cl. 1	Cl. 3	Cl. 1	Cl. 3	Cl. 1	Cl. 3	Cl. 1	Cl. 3	Cl. 1	Cl. 3	Cl. 1	Cl. 3
**Correctly Classified Instances (%)**	78.92	94.93	81.17	87.22	77.43	95.16	80.90	87.37	72.26	92.42	76.96	84.22
**Incorrectly Classified Instances (%)**	21.07	5.06	18.82	12.77	22.56	4.83	19.09	12.62	27.73	7.57	23.03	15.77
**Average Classification Accuracy (%)**	78.91	89.23	81.34	85.97	77.57	89.50	81.03	86.24	72.40	88.48	77.07	83.22
**Average Precision (%)**	78.82	90.67	81.15	86.88	77.42	91.30	80.87	86.98	72.27	84.26	76.94	83.44

**Table 4:** Numerical coding of the structure of analyzed chlorides.

Code	Condition Attributes	
	n-Spacer	R-Substituent
**1**		CH_3_
**2**	C_2_H_5_	C_2_H_5_
**3**	C_3_H_7_	C_3_H_7_
**4**	C_4_H_9_	C_4_H_9_
**5**	C_5_H_11_	C_5_H_11_
**6**	C_6_H_13_	C_6_H_13_
**7**	C_7_H_15_	C_7_H_15_
**8**	C_8_H_17_	C_8_H_17_
**9**	C_9_H_19_	C_9_H_19_
**10**	C_10_H_21_	C_10_H_21_
**11**		C_11_H_23_
**12**	C_12_H_25_	C_12_H_25_
**14**		C_14_H_29_
**16**		C_16_H_33_

**Table 5:** A part of the information system (15 from 140 objects).

No.	n	R	lgCMC	γCMC	G	A	G_ads_	MLOGP	BI	NTI	WI	MW	HOMO	LUMO	HL Gap	dip	ROG	TSC	MIC SAU (mM/L)	MIC PAE (mM/L)
**1**	2	1	2.15	61.9	2.75	52	20.2	0.175	1.397	12.712	5.275	252.36	−0.38777	−0.19852	−0.18925	1.646	4.908	0.28	16.937	33.875
**2**	2	2	2.23	60.1	2.71	54	20.8	0.711	1.407	14.099	5.768	280.42	−0.38416	−0.19108	−0.19308	0.103	5.294	0.266	3.558	28.468
**3**	2	3	2.38	59.8	2.69	56	21.3	1.216	1.405	15.485	6.307	308.48	−0.38269	−0.18871	−0.19398	2.314	5.804	0.254	3.295	13.181
**4**	2	4	2.41	57.4	2.65	58	21.7	1.697	1.397	16.871	6.877	336.54	−0.38194	−0.18751	−0.19443	5.474	6.246	0.243	1.634	13.180
**5**	2	5	2.49	55.5	2.61	60	22.3	2.157	1.386	18.257	7.468	364.6	−0.38150	−0.18679	−0.19471	8.628	6.777	0.234	0.712	11.483
**6**	2	6	2.58	53.4	2.57	62	22.7	2.599	1.373	19.644	8.074	392.66	−0.38544	−0.18641	−0.19903	12.501	7.25	0.226	0.086	10.788
**7**	2	7	2.65	51.2	2.53	64	23.5	3.025	1.359	21.03	8.692	420.72	−0.38109	−0.18618	−0.19491	16.201	7.791	0.218	0.020	5.086
**8**	2	8	2.72	48.9	2.49	66	23.9	4.349	1.346	22.416	9.319	448.78	−0.36560	−0.18605	−0.17955	20.472	8.282	0.211	0.005	1.193
**9**	2	9	2.81	47.5	2.45	68	24.3	4.748	1.333	23.803	9.952	476.84	−0.35218	−0.18590	−0.16628	24.499	8.831	0.205	0.002	1.132
**10**	2	10	2.92	45.3	2.41	70	24.8	5.136	1.32	25.189	10.59	504.9	−0.34105	−0.18584	−0.15521	29.011	9.333	0.199	0.017	0.538
**11**	2	11	3.04	42.5	2.37	72	25.6	5.514	1.308	26.575	11.233	532.96	−0.33153	−0.18577	−0.14576	33.258	9.883	0.194	0.017	0.513
**12**	2	12	3.15	41.4	2.33	74	26.3	5.883	1.297	27.961	11.879	561.02	−0.32343	−0.18573	−0.13770	37.935	10.392	0.189	0.016	0.491
**13**	2	14	3.34	37.5	2.25	78	27.5	6.595	1.277	30.734	13.18	617.14	−0.31025	−0.18566	−0.12459	47.138	11.454	0.18	0.116	1.817
**14**	2	16	3.52	33.9	2.17	82	28.8	7.278	1.26	33.507	14.488	673.26	−0.30006	−0.18563	−0.11443	56.553	12.515	0.173	0.108	1.680
**15**	3	1	2.18	60.8	2.74	54	20.5	0.447	1.363	13.405	5.649	266.39	−0.37891	−0.19219	−0.18672	1.941	5.192	0.273	29.652	29.652

## Supplementary Materials

The following are available online at https://www.mdpi.com/article/10.3390/ijms22157997/s1, Table S1: Information system.
