# Supplementary material for: Structure-Activity Relationships of the Imidazolium Compounds as Antibacterials of Staphylococcus aureus and Pseudomonas aeruginosa
Source: Int J Mol Sci. 2021 Jul 27;22(15):7997. doi: 10.3390/ijms22157997 (PMC8347611; doi:10.3390/ijms22157997)
Supplement: Supplementary file 1 [file ijms-22-07997-s001.zip › ijms-1288988-supplementary.pdf]

International Journal of *Molecular Sciences*

MDPI

Structure−Activity Relationships of the Imidazolium Compounds as Antibacterials of *Staphylococcus aureus* and *Pseudomonas aeruginosa*

Łukasz Pałkowski [1*], Maciej Karolak [1], Jerzy Błaszczyński [2], Jerzy Krysiński [1] and Roman Słowiński [2,3]

Table 1. Information system.

Object	n	R	logCMC	gCMC	G	A	Gads	MLOGP	Balaban index	Narumi topological index	MW	HOMO	LUMO	HOMO–LUMO gap	dipole	Radius of Gyration	total structure connectivity index	Wiener index	SAU	PAE
1	2	1	2,15	61,9	2,75	52	20,2	0,175	1,397	12,712	252,36	−0,38777	−0,19852	−0,18925	1,646	4,908	0,28	5,275	30,95975	33,87500
2	2	2	2,23	60,1	2,71	54	20,8	0,711	1,407	14,099	280,42	−0,38416	−0,19108	−0,19308	0,103	5,294	0,266	5,768	28,46797	28,46800
3	2	3	2,38	59,8	2,69	56	21,3	1,216	1,405	15,485	308,48	−0,38269	−0,18871	−0,19398	2,314	5,804	0,254	6,307	13,18131	13,18100
4	2	4	2,41	57,4	2,65	58	21,7	1,697	1,397	16,871	336,54	−0,38194	−0,18751	−0,19443	5,474	6,246	0,243	6,877	6,14251	13,18000
5	2	5	2,49	55,5	2,61	60	22,3	2,157	1,386	18,257	364,6	−0,38150	−0,18679	−0,19471	8,628	6,777	0,234	7,468	5,74144	11,48300
6	2	6	2,58	53,4	2,57	62	22,7	2,599	1,373	19,644	392,66	−0,38544	−0,18641	−0,19903	12,501	7,25	0,226	8,074	1,33769	10,78800
7	2	7	2,65	51,2	2,53	64	23,5	3,025	1,359	21,03	420,72	−0,38109	−0,18618	−0,19491	16,201	7,791	0,218	8,692	0,16275	5,08600
8	2	8	2,72	48,9	2,49	66	23,9	4,349	1,346	22,416	448,78	−0,36560	−0,18605	−0,17955	20,472	8,282	0,211	9,319	0,03849	1,19300
9	2	9	2,81	47,5	2,45	68	24,3	4,748	1,333	23,803	476,84	−0,35218	−0,18590	−0,16628	24,499	8,831	0,205	9,952	0,01826	1,13200
10	2	10	2,92	45,3	2,41	70	24,8	5,136	1,32	25,189	504,9	−0,34105	−0,18584	−0,15521	29,011	9,333	0,199	10,59	0,01737	0,53800
11	2	11	3,04	42,5	2,37	72	25,6	5,514	1,308	26,575	532,96	−0,33153	−0,18577	−0,14576	33,258	9,883	0,194	11,233	0,01656	0,51300
12	2	12	3,15	41,4	2,33	74	26,3	5,883	1,297	27,961	561,02	−0,32343	−0,18573	−0,13770	37,935	10,392	0,189	11,879	0,03166	0,49100
13	2	14	3,34	37,5	2,25	78	27,5	6,595	1,277	30,734	617,14	−0,31025	−0,18566	−0,12459	47,138	11,454	0,18	13,18	0,05815	1,81700
14	2	16	3,52	33,9	2,17	82	28,8	7,278	1,26	33,507	673,26	−0,30006	−0,18563	−0,11443	56,553	12,515	0,173	14,488	0,10753	1,68000
15	3	1	2,18	60,8	2,74	54	20,5	0,447	1,363	13,405	266,39	−0,37891	−0,19219	−0,18672	1,941	5,192	0,273	5,649	29,65203	29,65200
16	3	2	2,26	58,9	2,7	56	21,1	0,967	1,374	14,792	294,45	−0,37602	−0,18624	−0,18978	2,076	5,608	0,26	6,143	27,37484	27,37500
17	3	3	2,32	57,8	2,66	58	21,6	1,46	1,375	16,178	322,51	−0,37432	−0,18443	−0,18989	2,426	6,115	0,249	6,68	25,42252	25,42300
18	3	4	2,44	56,3	2,62	60	22	1,929	1,37	17,564	350,57	−0,37438	−0,18333	−0,19105	3,437	6,617	0,239	7,247	5,93254	5,93300
19	3	5	2,52	54,4	2,58	62	22,6	2,38	1,362	18,951	378,63	−0,37327	−0,18255	−0,19072	4,782	7,183	0,23	7,835	5,56226	22,24900

20	3	6	2,61	52,3	2,54	64	23	2,814	1,351	20,337	406,69	−0,37302	−0,18221	−0,19081	6,850	7,726	0,222	8,438	1,29840	10,47100
21	3	7	2,68	50,1	2,59	66	23,8	3,232	1,34	21,723	434,75	−0,37321	−0,18137	−0,19184	9,525	8,348	0,215	9,054	0,15824	4,94500
22	3	8	2,75	47,9	2,46	68	24,2	4,55	1,329	23,109	462,81	−0,36030	−0,18122	−0,17908	12,379	8,912	0,208	9,678	0,00937	1,16200
23	3	9	2,84	46,4	2,42	70	24,6	4,944	1,318	24,496	490,87	−0,34773	−0,18113	−0,16660	14,885	9,513	0,202	10,309	0,06944	4,45100
24	3	10	2,95	44,2	2,38	72	25,1	5,327	1,307	25,882	518,93	−0,33649	−0,18116	−0,15533	17,874	10,09	0,197	10,946	0,00848	0,52600
25	3	11	3,07	42,4	2,34	74	25,9	5,7	1,297	27,268	546,99	−0,32734	−0,18055	−0,14679	22,854	10,668	0,192	11,587	0,01619	0,50200
26	3	12	3,18	40,3	2,3	76	22,6	6,064	1,287	28,655	575,05	−0,31947	−0,18057	−0,13890	26,450	11,24	0,187	12,232	0,00774	15,48400
27	3	14	3,37	36,4	2,22	80	27,7	6,769	1,269	31,427	631,17	−0,30693	−0,18170	−0,12523	24,431	12,739	0,178	13,529	0,05556	7,12300
28	3	16	3,55	32,8	2,14	84	29,1	7,445	1,252	34,2	687,29	−0,29728	−0,18211	−0,11517	19,258	14,623	0,171	14,835	0,10554	13,19200
29	4	1	2,22	59,6	2,71	56	20,9	0,711	1,334	14,099	280,42	−0,36306	−0,18370	−0,17936	1,568	5,744	0,266	6,021	28,46797	28,46800
30	4	2	2,3	57,7	2,67	58	21,4	1,216	1,346	15,485	308,48	−0,35990	−0,17667	−0,18323	0,100	6,137	0,254	6,515	6,59065	26,36300
31	4	3	2,39	56,6	2,63	60	21,9	1,697	1,349	16,871	336,54	−0,35870	−0,17437	−0,18433	1,934	6,639	0,243	7,051	12,28501	26,36000
32	4	4	2,45	55,1	2,59	62	22,3	2,157	1,346	18,257	364,6	−0,35806	−0,17325	−0,18481	4,875	7,075	0,234	7,615	0,71194	11,48300
33	4	5	2,53	53,2	2,55	64	22,9	2,599	1,34	19,644	392,66	−0,35770	−0,17257	−0,18513	7,849	7,597	0,226	8,201	0,34521	10,78800
34	4	6	2,64	51,1	2,51	66	23,3	3,025	1,332	21,03	420,72	−0,35747	−0,17227	−0,18520	11,527	8,063	0,218	8,802	0,07934	5,08600
35	4	7	2,71	48,9	2,47	68	23,9	3,437	1,323	22,416	448,78	−0,35989	−0,17502	−0,18487	12,089	8,72	0,211	9,415	0,00962	1,19300
36	4	8	2,79	46,7	2,43	70	24,5	4,748	1,314	23,803	476,84	−0,35824	−0,17488	−0,18336	15,485	9,261	0,205	10,037	0,00913	2,28300
37	4	9	2,88	45,2	2,39	72	24,9	5,136	1,304	25,189	504,9	−0,34550	−0,17481	−0,17069	18,773	9,836	0,199	10,667	0,00869	0,27800
38	4	10	3	43	2,35	74	25,4	5,514	1,294	26,575	532,96	−0,33482	−0,17454	−0,16028	22,419	10,401	0,194	11,302	0,00828	0,06600
39	4	11	3,13	41,2	2,31	76	26,2	5,883	1,285	27,961	561,02	−0,32570	−0,17379	−0,15191	25,720	11,008	0,189	11,941	0,03166	3,95700
40	4	12	3,22	39,2	2,27	78	26,9	6,243	1,277	29,348	589,08	−0,31806	−0,17375	−0,14431	29,909	11,545	0,185	12,584	0,06062	0,94000
41	4	14	3,4	35,2	2,19	82	28	6,94	1,26	32,12	645,2	−0,30562	−0,17364	−0,13198	37,930	12,669	0,176	13,879	0,02793	0,22300
42	4	16	3,58	31,5	2,11	86	29,3	7,61	1,246	34,893	701,32	−0,29602	−0,17361	−0,12241	46,251	13,792	0,169	15,183	0,05181	12,95200
43	5	1	2,25	58,5	2,68	58	21,2	0,967	1,309	14,792	294,45	−0,35499	−0,17874	−0,17625	1,248	6,108	0,26	6,39	27,37484	27,37500
44	5	2	2,33	56,6	2,64	60	21,7	1,46	1,322	16,178	322,51	−0,35210	−0,17344	−0,17866	0,553	6,604	0,249	6,885	25,42252	25,42300
45	5	3	2,41	55,7	2,6	62	22,2	1,929	1,326	17,564	350,57	−0,35085	−0,17131	−0,17954	0,611	7,159	0,239	7,42	5,93254	23,73000
46	5	4	2,49	53,9	2,56	64	22,7	2,38	1,325	18,951	378,63	−0,35017	−0,17022	−0,17995	1,120	7,748	0,23	7,983	0,17799	11,12500

47	5	5	2,58	52,1	2,52	66	23,2	2,814	1,321	20,337	406,69	−0,34977	−0,16970	−0,18007	2,184	8,358	0,222	8,567	2,61774	20,94200
48	5	6	2,67	50,1	2,48	68	23,7	3,232	1,315	21,723	434,75	−0,34954	−0,16942	−0,18012	2,510	8,996	0,215	9,166	0,15824	2,47200
49	5	7	2,75	47,8	2,44	70	24,2	3,638	1,307	23,109	462,81	−0,34938	−0,16935	−0,18003	3,487	9,629	0,208	9,777	0,01874	18,74000
50	5	8	2,83	45,6	2,4	72	24,8	4,944	1,299	24,496	490,87	−0,34931	−0,16925	−0,18006	4,040	10,294	0,202	10,397	0,14243	2,22600
51	5	9	2,91	44	2,36	74	25,3	5,327	1,291	25,882	518,93	−0,34372	−0,16922	−0,17450	4,922	10,96	0,197	11,024	0,00848	0,13600
52	5	10	3,07	41,9	2,32	76	25,9	5,7	1,283	27,268	546,99	−0,33324	−0,16918	−0,16406	5,563	11,647	0,192	11,657	0,00405	0,06500
53	5	11	3,16	40,1	2,28	78	26,5	6,064	1,275	28,655	575,05	−0,32440	−0,16922	−0,15518	6,612	12,318	0,187	12,295	0,01548	0,24800
54	5	12	3,25	38,1	2,24	80	27,1	6,42	1,267	30,041	603,11	−0,31653	−0,16893	−0,14760	6,932	13,109	0,182	12,937	0,02968	0,46000
55	5	14	3,43	33,9	2,16	84	28,3	7,11	1,252	32,813	659,23	−0,30493	−0,17155	−0,13338	26,669	13,391	0,175	14,229	0,02740	0,84900
56	5	16	3,61	29,6	2,08	88	29,6	7,773	1,239	35,586	715,35	−0,29534	−0,17122	−0,12412	32,878	14,639	0,168	15,531	0,20354	12,72100
57	6	1	2,29	47,1	2,73	56	29,3	1,216	1,288	15,485	308,48	−0,34837	−0,17532	−0,17305	1,779	6,552	0,254	6,758	6,59065	26,36300
58	6	2	2,37	44,8	2,66	59	30,2	1,697	1,301	16,871	336,54	−0,34558	−0,16970	−0,17588	0,225	6,977	0,243	7,254	0,76167	26,36000
59	6	3	2,45	42,6	2,59	62	30,9	2,157	1,306	18,257	364,6	−0,34461	−0,16569	−0,17892	1,613	7,477	0,234	7,788	0,71194	22,96600
60	6	4	2,54	39,4	2,53	65	31,5	2,599	1,306	19,644	392,66	−0,34396	−0,16457	−0,17939	4,338	7,912	0,226	8,349	0,66885	2,69700
61	6	5	2,62	37,6	2,47	68	32,1	3,025	1,304	21,03	420,72	−0,34360	−0,16398	−0,17962	7,165	8,42	0,218	8,931	0,04069	5,08600
62	6	6	2,71	36,3	2,41	71	32,6	3,437	1,299	22,416	448,78	−0,34341	−0,16367	−0,17974	10,654	8,883	0,211	9,528	0,07698	1,19300
63	6	7	2,79	35,1	2,34	74	33,1	3,836	1,293	23,803	476,84	−0,34328	−0,16346	−0,17982	14,068	9,407	0,205	10,137	0,01826	0,56600
64	6	8	2,87	34,2	2,28	77	33,6	5,136	1,286	25,189	504,9	−0,34323	−0,16329	−0,17994	17,954	9,899	0,199	10,756	0,00869	1,07700
65	6	9	2,95	33,3	2,22	80	34	5,514	1,279	26,575	532,96	−0,34316	−0,16323	−0,17993	21,759	10,429	0,194	11,381	0,01656	0,03300
66	6	10	3,07	32,2	2,15	83	34,4	5,883	1,272	27,961	561,02	−0,33468	−0,16313	−0,17155	25,938	10,928	0,189	12,013	0,00396	0,01600
67	6	11	3,2	31,2	2,09	86	34,8	6,243	1,265	29,348	589,08	−0,32570	−0,16312	−0,16258	29,995	11,466	0,185	12,649	0,01516	0,47000
68	6	12	3,29	30,3	2,03	89	35,2	6,595	1,258	30,734	617,14	−0,31808	−0,16310	−0,15498	34,413	11,97	0,18	13,29	0,02907	3,63400
69	6	14	3,47	28,3	1,9	95	36,1	7,278	1,245	33,507	673,26	−0,30573	−0,16306	−0,14267	43,206	13,017	0,173	14,58	0,02688	0,41700
70	6	16	3,65	26,4	1,77	101	37,1	7,935	1,233	36,279	729,38	−0,29621	−0,16304	−0,13317	52,243	14,071	0,166	15,879	0,09999	1,56200
71	7	1	2,27	50,3	2,61	60	27,2	1,46	1,269	16,178	322,51	−0,34321	−0,17182	−0,17139	2,967	6,942	0,249	7,123	12,71126	33,87476
72	7	2	2,38	48,5	2,58	62	28,1	1,929	1,282	17,564	350,57	−0,34052	−0,16474	−0,17578	1,267	7,334	0,239	7,62	0,73563	28,46797
73	7	3	2,48	46,7	2,55	64	29,1	2,38	1,288	18,951	378,63	−0,33959	−0,16412	−0,17547	0,084	8,007	0,23	8,154	0,35598	13,18131

74	7	4	2,57	44,9	2,52	66	30,2	2,814	1,29	20,337	406,69	−0,33899	−0,16304	−0,17595	0,462	8,594	0,222	8,714	0,08377	26,35984
75	7	5	2,68	43,1	2,49	68	31,2	3,232	1,288	21,723	434,75	−0,33862	−0,16252	−0,17610	1,518	9,202	0,215	9,295	0,03956	2,87072
76	7	6	2,78	41,3	2,46	70	32,3	3,638	1,285	23,109	462,81	−0,33842	−0,16224	−0,17618	1,889	9,836	0,208	9,89	0,00469	1,33769
77	7	7	2,89	39,5	2,43	72	33,2	4,032	1,28	24,496	490,87	−0,33822	−0,16223	−0,17599	2,917	10,448	0,202	10,497	0,00890	0,32551
78	7	8	2,99	37,7	2,4	74	34,3	5,327	1,274	25,882	518,93	−0,33814	−0,16217	−0,17597	3,562	11,101	0,197	11,114	0,00212	0,30793
79	7	9	3,09	35,9	2,37	76	35,4	5,7	1,268	27,268	546,99	−0,33811	−0,16206	−0,17605	4,427	11,781	0,192	11,738	0,00202	0,29216
80	7	10	3,19	33,1	2,34	78	36,3	6,064	1,262	28,655	575,05	−0,33088	−0,16204	−0,16884	4,882	12,455	0,187	12,368	0,00194	0,06948
81	7	11	3,29	31,3	2,31	80	37,3	6,42	1,256	30,041	603,11	−0,32234	−0,16221	−0,16013	26,879	12,034	0,182	13,003	0,00371	0,26501
82	7	12	3,4	29,6	2,26	82	38,2	6,769	1,25	31,427	631,17	−0,31485	−0,16217	−0,15268	30,572	12,572	0,178	13,642	0,00712	0,49066
83	7	14	3,51	27,8	2,19	86	40,3	7,445	1,238	34,2	687,29	−0,30296	−0,16230	−0,14066	38,959	13,525	0,171	14,93	0,00660	0,90128
84	7	16	3,62	26,1	2,11	90	42,2	8,096	1,227	36,972	743,41	−0,29361	−0,16225	−0,13136	46,727	14,629	0,164	16,228	0,01228	1,68007
85	8	1	2,25	56,2	2,51	63	19,8	1,697	1,253	16,871	336,54	−0,33972	−0,16794	−0,17178	1,275	7,43	0,243	7,486	0,21088	1,83843
86	8	2	2,36	54,1	2,47	65	20,7	2,157	1,266	18,257	364,6	−0,33642	−0,16198	−0,17444	0,158	7,806	0,234	7,985	0,18373	3,42185
87	8	3	2,45	52,1	2,44	67	21,5	2,599	1,272	19,644	392,66	−0,33533	−0,16116	−0,17417	1,278	8,25	0,226	8,519	0,00539	1,57620
88	8	4	2,49	50,2	2,41	69	22,3	3,025	1,275	21,03	420,72	−0,33483	−0,16023	−0,17460	3,295	8,626	0,218	9,078	0,32551	5,93254
89	8	5	2,55	48,3	2,37	71	23,1	3,437	1,274	22,416	448,78	−0,33571	−0,16073	−0,17498	4,862	9,244	0,211	9,657	0,07698	2,78113
90	8	6	2,64	46,3	2,35	73	23,9	3,836	1,272	23,803	476,84	−0,33552	−0,16047	−0,17505	7,500	9,743	0,205	10,251	0,03652	0,33507
91	8	7	2,71	44,2	2,33	75	24,8	4,224	1,268	25,189	504,9	−0,33541	−0,16032	−0,17509	10,171	10,276	0,199	10,857	0,00869	0,31648
92	8	8	2,9	42,1	2,31	77	25,6	5,514	1,263	26,575	532,96	−0,33547	−0,15982	−0,17565	13,350	10,85	0,194	11,472	0,00207	0,29984
93	8	9	2,98	39,7	2,28	79	26,8	5,883	1,258	27,961	561,02	−0,33541	−0,15978	−0,17563	16,618	11,397	0,189	12,095	0,00396	0,55192
94	8	10	3,08	37,6	2,25	81	27,3	6,243	1,253	29,348	589,08	−0,33011	−0,15972	−0,17039	20,080	11,935	0,185	12,724	0,00379	0,03391
95	8	11	3,17	35,4	2,22	83	27,9	6,595	1,247	30,734	617,14	−0,32143	−0,15970	−0,16173	23,435	12,496	0,18	13,357	0,01454	0,12950
96	8	12	3,28	33,1	2,19	85	29,1	6,94	1,242	32,12	645,2	−0,31400	−0,15953	−0,15447	27,003	13,059	0,176	13,995	0,01397	1,93549
97	8	14	3,45	29,1	2,12	89	30,8	7,61	1,231	34,893	701,32	−0,30209	−0,15925	−0,14284	34,708	14,162	0,169	15,281	0,01295	3,56158
98	8	16	3,69	25,4	2,04	93	32,5	8,255	1,221	37,666	757,44	−0,29297	−0,15913	−0,13384	42,979	15,248	0,163	16,577	0,04830	1,64898
99	9	1	2,21	50,1	2,44	67	28,6	1,929	1,239	17,564	350,57	−0,33790	−0,16436	−0,17354	1,569	7,839	0,239	7,847	0,37968	28,46797
100	9	2	2,27	49,1	2,41	69	28,9	2,38	1,251	18,951	378,63	−0,33325	−0,16118	−0,17207	1,894	8,291	0,23	8,348	0,08900	26,36262

101	9	3	2,33	48,2	2,37	71	29,3	2,814	1,258	20,337	406,69	−0,33220	−0,15906	−0,17314	0,707	8,847	0,222	8,882	0,08377	26,35984
102	9	4	2,39	47,1	2,34	73	29,6	3,232	1,261	21,723	434,75	−0,33163	−0,15799	−0,17364	0,124	9,434	0,215	9,441	0,01978	2,87072
103	9	5	2,45	46,1	2,31	75	29,9	3,638	1,261	23,109	462,81	−0,33131	−0,15746	−0,17385	0,909	10,04	0,208	10,019	0,00937	1,33769
104	9	6	2,51	45,1	2,29	77	31,3	4,032	1,26	24,496	490,87	−0,33112	−0,15718	−0,17394	1,318	10,671	0,202	10,612	0,08902	0,63067
105	9	7	2,56	44	2,27	79	31,6	4,415	1,257	25,882	518,93	−0,33091	−0,15724	−0,17367	2,306	11,267	0,197	11,216	0,00424	1,19325
106	9	8	2,61	42,9	2,24	81	31,9	5,7	1,253	27,268	546,99	−0,33086	−0,15710	−0,17376	2,574	11,935	0,192	11,83	0,00809	0,56606
107	9	9	2,66	41,8	2,21	83	32,3	6,064	1,249	28,655	575,05	−0,33081	−0,15711	−0,17370	3,598	12,589	0,187	12,451	0,01548	1,07696
108	9	10	2,72	40,6	2,18	85	32,7	6,42	1,244	30,041	603,11	−0,32897	−0,15701	−0,17196	5,172	13,232	0,182	13,079	0,02968	0,26501
109	9	11	2,78	39,5	2,15	87	33,1	6,769	1,239	31,427	631,17	−0,32049	−0,15715	−0,16334	24,420	12,782	0,178	13,711	0,01425	0,98132
110	9	12	2,84	38,4	2,11	89	33,5	7,11	1,234	32,813	659,23	−0,31314	−0,15706	−0,15608	27,688	13,315	0,175	14,348	0,05480	1,89435
111	9	14	2,98	36,2	2,02	93	34,4	7,773	1,225	35,586	715,35	−0,30129	−0,15708	−0,14421	34,938	14,398	0,168	15,631	0,05088	1,74590
112	9	16	3,11	34,1	1,92	97	35,1	8,413	1,215	38,359	771,47	−0,29208	−0,15671	−0,13537	37,453	15,959	0,161	16,925	0,02375	0,20724
113	10	1	2,18	44,1	2,37	73	31,4	2,157	1,226	18,257	364,6	−0,33504	−0,16223	−0,17281	0,992	8,28	0,234	8,206	0,18373	27,37484
114	10	2	2,23	43,2	2,35	74	31,6	2,599	1,238	19,644	392,66	−0,33211	−0,15568	−0,17643	0,415	8,75	0,226	8,709	0,17261	25,42252
115	10	3	2,29	42,2	2,33	75	31,8	3,025	1,245	21,03	420,72	−0,33015	−0,15789	−0,17226	0,692	9,055	0,218	9,244	0,08138	11,86507
116	10	4	2,33	41,2	2,31	76	32	3,437	1,249	22,416	448,78	−0,32961	−0,15680	−0,17281	2,332	9,546	0,211	9,802	0,03849	22,24902
117	10	5	2,4	40,3	2,29	77	32,2	3,836	1,25	23,803	476,84	−0,32931	−0,15627	−0,17304	4,405	10,055	0,205	10,38	0,00057	0,64920
118	10	6	2,46	39,2	2,27	78	32,4	4,224	1,249	25,189	504,9	−0,32912	−0,15595	−0,17317	6,725	10,586	0,199	10,971	0,00109	0,61318
119	10	7	2,51	38,4	2,25	79	32,6	4,602	1,247	26,575	532,96	−0,32903	−0,15584	−0,17319	9,444	11,082	0,194	11,575	0,00414	0,58094
120	10	8	2,56	37,5	2,23	80	32,8	5,883	1,244	27,961	561,02	−0,32896	−0,15577	−0,17319	12,466	11,6	0,189	12,187	0,01583	0,55192
121	10	9	2,61	36,5	2,21	81	33	6,243	1,24	29,348	589,08	−0,32877	−0,15470	−0,17407	14,602	12,497	0,185	12,807	0,00758	1,05134
122	10	10	2,66	35,6	2,19	82	33,2	6,595	1,236	30,734	617,14	−0,32831	−0,15462	−0,17369	17,753	13,046	0,18	13,433	0,02907	0,25899
123	10	11	2,72	34,6	2,17	83	33,4	6,94	1,232	32,12	645,2	−0,31933	−0,15421	−0,16512	19,171	13,736	0,176	14,065	0,02793	0,96000
124	10	12	2,78	33,5	2,15	84	33,6	7,278	1,227	33,507	673,26	−0,31198	−0,15425	−0,15773	22,463	14,296	0,173	14,7	0,02688	7,41969
125	10	14	2,91	31,4	2,11	86	34,1	7,935	1,219	36,279	729,38	−0,30022	−0,15438	−0,14584	28,675	15,453	0,166	15,982	0,02500	1,71235
126	10	16	3,04	29,3	2,07	88	34,6	8,57	1,21	39,052	785,5	−0,29119	−0,15451	−0,13668	35,127	16,618	0,16	17,274	0,02336	6,36055
127	12	1	2,29	43,6	2,23	76	31,9	2,599	1,205	19,644	392,66	−0,33072	−0,15874	−0,17198	0,859	9,103	0,226	8,921	0,04315	26,36262

128	12	2	2,34	42,5	2,21	77	32,1	3,025	1,216	21,03	420,72	−0,33806	−0,15358	−0,18448	0,381	9,566	0,218	9,428	0,08138	26,35984
129	12	3	2,4	41,7	2,19	78	32,3	3,437	1,223	22,416	448,78	−0,32658	−0,15191	−0,17467	1,114	10,077	0,211	9,964	0,01925	5,74144
130	12	4	2,46	41,4	2,17	79	32,5	3,836	1,227	23,803	476,84	−0,32584	−0,15139	−0,17445	2,049	10,627	0,205	10,522	0,00913	2,69696
131	12	5	2,51	39,9	2,15	80	32,7	4,224	1,229	25,189	504,9	−0,32523	−0,15108	−0,17415	3,514	11,187	0,199	11,098	0,00109	2,54304
132	12	6	2,56	39,1	2,13	81	32,9	4,602	1,229	26,575	532,96	−0,32501	−0,15086	−0,17415	5,381	11,726	0,194	11,688	0,00828	1,19325
133	12	7	2,62	38,1	2,11	82	33,1	4,971	1,228	27,961	561,02	−0,32481	−0,15079	−0,17402	7,255	12,3	0,189	12,29	0,00050	0,56606
134	12	8	2,67	37,2	2,09	83	33,3	6,243	1,226	29,348	589,08	−0,32469	−0,15076	−0,17393	9,512	12,852	0,185	12,9	0,01516	0,27792
135	12	9	2,73	36,2	2,07	84	33,5	6,595	1,224	30,734	617,14	−0,32458	−0,15075	−0,17383	11,727	13,435	0,18	13,518	0,00363	4,14079
136	12	10	2,78	35,5	2,05	85	33,7	6,94	1,221	32,12	645,2	−0,32444	−0,15080	−0,17364	15,311	13,978	0,176	14,142	0,01397	1,97846
137	12	11	2,85	34,5	2,03	86	33,9	7,278	1,218	33,507	673,26	−0,31799	−0,15084	−0,16715	17,977	14,565	0,173	14,771	0,05376	3,78870
138	12	12	2,89	33,9	2,01	87	34,1	7,61	1,214	34,893	701,32	−0,31079	−0,15083	−0,15996	21,014	15,137	0,169	15,405	0,05181	14,53679
139	12	14	3,01	32,4	1,97	89	34,5	8,255	1,207	37,666	757,44	−0,29910	−0,15096	−0,14814	27,328	16,286	0,163	16,683	0,04830	6,72029
140	12	16	3,12	30,9	1,93	91	34,9	8,88	1,2	40,438	813,56	−0,29018	−0,15109	−0,13909	33,603	17,445	0,157	17,972	0,09047	6,24904
